# Fresh ginseng-derived vesicle-like nanoparticles protect against intestinal barrier injury in inflammatory bowel disease via the miR-29a-5p/TLR4 axis

**DOI:** 10.3389/fimmu.2026.1929376

**Published:** 2026-09-30

**Authors:** Xiaojuan Tang, Yuan Ren, Min He, Yuan Lv, Ning Ding, Fang Liu, Yongheng He, Yongmin Li

**Affiliations:** 1 Central Laboratory, Hunan Provincial Hospital of Integrated Traditional Chinese and Western Medicine (The Affiliated Hospital of Hunan Academy of Traditional Chinese Medicine), Changsha, Hunan, China; 2 Graduate School, Hunan University of Chinese Medicine, Changsha, Hunan, China; 3 Department of Anorectal Diseases, Second Affiliated Hospital of Hunan University of Chinese Medicine, Changsha, Hunan, China

**Keywords:** ginseng-derived vesicle-like nanoparticles, inflammatory bowel disease, intestinal barrier, macrophage polarization, miR-29a-5p/TLR4

## Abstract

**Background:**

Inflammatory bowel disease (IBD) remains therapeutically challenging, and disruption of the intestinal epithelial barrier constitutes a key pathogenic event. Ginseng-derived vesicle-like nanoparticles (GDNPs) protect against intestinal barrier injury in IBD; however, the effects of ginseng processing on nanoparticle characteristics and biological activity remain insufficiently understood.

**Methods:**

The protective effect of fresh and dried ginseng extract were compared in IBD model. GDNPs were isolated from fresh and dried ginseng by differential centrifugation and sucrose density-gradient ultracentrifugation, and characterized for morphology, particle size, and relative purity. Their effects on intestinal epithelial cell viability and permeability were compared. *In vitro*, inflamed NCM460 and RAW264.7 cells were treated with fresh GDNPs; tight junction protein expression and TLR4/STAT3 signaling were assessed by western blotting, and cytokine and miRNA (miR-29a-5p, miR-125b-5p, miR-21-5p) levels were measured by RT-qPCR. Trans-epithelial electrical resistance (TEER) was measured in epithelial cells overexpressing miR-29a-5p, and tight junction protein and TLR4 expression were examined after miR-29a-5p knockdown; direct targeting of TLR4 by miR-29a-5p was confirmed by dual-luciferase reporter assay. Intestinal epithelial cells were also exposed to conditioned medium from fresh GDNP-treated macrophages to assess tight junction protein and miR-29a-5p expression. *In vivo*, a dextran sulfate sodium (DSS)-induced murine colitis model was used to evaluate the preventive protective effects of low- or high-dose fresh GDNPs, and body weight, disease activity index (DAI), colon length, histopathology, macrophage polarization, tight junction protein expression, and inflammatory cytokine and miR-29a-5p levels were evaluated.

**Results:**

Both fresh and dried ginseng extracts alleviated DSS-induced intestinal injury, with fresh ginseng extract showing stronger protective effects in several pathological and inflammatory parameters. Fresh and dried GDNPs showed comparable spherical morphology and particle-to-protein ratios, but fresh GDNPs were more susceptible to Triton X-100-induced lysis, indicating a higher proportion of intact vesicles. Fresh GDNPs promoted intestinal epithelial cell viability, whereas dried GDNPs at the same concentration had no significant effect, and fresh GDNPs conferred stronger protection against epithelial permeability disruption. In inflamed epithelial and macrophage models, fresh GDNPs suppressed TLR4/STAT3 signaling and pro-inflammatory cytokines (IL-6, IL-1β, TNF-α) while restoring tight junction proteins (Occludin, Claudin-1) and miR-29a-5p expression. Mechanistically, fresh GDNPs preserved epithelial barrier integrity by upregulating miR-29a-5p, which directly targeted and suppressed TLR4, and by reprogramming the macrophage inflammatory secretome. In DSS-induced colitis, fresh GDNPs attenuated body weight loss, DAI scores, and colon shortening; reduced M1 macrophage polarization; preserved tight junction protein expression; and restored miR-29a-5p expression in colon tissue.

**Conclusion:**

Fresh and dried ginseng-derived GDNPs exhibited differential epithelial protective effects *in vitro*. Fresh ginseng-derived GDNPs protected intestinal barrier integrity through the miR-29a-5p/TLR4 axis and macrophage–epithelial crosstalk.

## 1 Introduction

Inflammatory bowel disease (IBD), comprising Crohn’s disease and ulcerative colitis, is a chronic relapsing–remitting inflammatory disorder of the gastrointestinal tract with multifactorial etiology (1). Its incidence and prevalence are rising rapidly in China, positioning IBD as an emerging public health priority with substantial socioeconomic burden (2). Compromised intestinal barrier function is a central pathophysiological axis driving IBD progression. Upon exposure to luminal pathogens, activated macrophages initiate a cytokine cascade dominated by tumor necrosis factor-α (TNF-α), interleukin-6 (IL-6), and interleukin-1β (IL-1β); these cytokines, in turn, directly downregulate tight junction proteins such as occludin and ZO-1 (3). The resulting paracellular permeability defect underlies the clinically recognized “leaky gut” phenomenon that characterizes most active IBD cases (4).

MicroRNAs (miRNAs), small noncoding RNAs of approximately 19–25 nucleotides, are key epigenetic regulators of intestinal barrier function and inflammation in IBD (5). Among them, members of the miR-29 family exert distinctive regulatory effects on hub genes and transcriptional networks relevant to epithelial homeostasis (6). For instance, miR-29b maintains intestinal epithelial homeostasis through competitive interaction with circHIPK3 (7), and miR-29a/b1-knockout mice display markedly aggravated inflammation, increased lymphocyte infiltration, altered macrophage polarization, and excessive extracellular matrix deposition that persists even during the tissue recovery phase (8). These observations establish miR-29 as both a disease mediator and a candidate therapeutic target for inflammation-driven epithelial repair, yet strategies to therapeutically restore miR-29 activity in IBD remain limited.

Ginseng (*Panax ginseng* C.A. Meyer), a classic herbal medicine, has recently been shown to yield ginseng-derived vesicle-like nanoparticles (GDNPs)—naturally occurring nanovesicles that encapsulate lipids, functional proteins, and miRNAs (9). GDNPs exert multiple beneficial effects relevant to IBD, including anti-inflammatory activity via TLR4/MAPK and p62/Keap1/Nrf2 signaling, modulation of the gut microbiota, and protection of the intestinal barrier (9–11). However, two important questions remain unresolved. First, although GDNPs can be extracted from either fresh or dried ginseng, prior studies have used fresh ginseng almost exclusively, and whether the choice of raw material affects nanoparticle quality and barrier-protective efficacy—an issue with direct relevance to the standardization and industrial scale-up of plant-derived nanoparticle therapeutics—has not been examined. Second, the molecular link between GDNPs-induced miRNA changes and the inflammatory TLR4 pathway, and the contribution of macrophage–epithelial crosstalk to this process, has not been mechanistically defined.

To address these gaps, this study first compared the protective effects of fresh and dried ginseng extracts in DSS induced colitis and subsequently investigated fresh ginseng derived GDNPs regarding their physicochemical characteristics, epithelial protective activity, and underlying mechanisms.

## 2 Materials and methods

### 2.1 Preparation of fresh and dried liquid extract from ginseng

Fresh ginseng roots (1 kg, obtained from Changbaishan, Jilin, China) were cut into 2-4mm pieces and homogenized in pre-chilled 1×PBS (1.5 L) using a blender equipped with a cooling jacket (4°C). The supernatants were collected and sequentially centrifuged at 500 × g for 5 min, 1,000 × g for 10 min, 2,000 × g for 10 min. Then the supernatant was concentrated by rotary evaporation to obtain the fresh extract.

Fresh ginseng roots (1 kg, obtained from Changbaishan, Jilin, China) were cut into 2–4-mm pieces, and the pieces were pre-frozen at –30°C for 2 h, then freeze‐dried at –10°C, 70 KPa for 8 h. The dried product was powdered, extracted twice with 10× distilled water, and the pooled extract was concentrated by rotary evaporation to obtain the dried extract.

### 2.2 Isolation and purification of GDNPs from fresh and dried ginseng

Supernatants from fresh ginseng pieces were collected and sequentially centrifuged at 800 × g for 5 min, 2,000 × g for 5 min, and 10,000 × g for 30 min. The resulting supernatant was then subjected to ultracentrifugation (100,000 × g, 1 h, 4°C), and the pellet containing nanoparticles was resuspended in ice-cold 1× PBS.

For dried ginseng pieces, they were soaked in ice-cold 1× PBS overnight and then homogenized. The homogenate was centrifuged in the same manner as the fresh samples, and the fresh and dried supernatants were layered onto a discontinuous sucrose gradient (15%, 30%, 45%, 60% w/v) and ultracentrifuged (100,000 × g, 1 h, 4°C). The fraction enriched with GDNPs at the 30–45% interface was collected and washed three times with PBS.

### 2.3 Characterization of GDNPs

Nanostructures were visualized by transmission electron microscopy (TEM, JEOL JEM-1400) at 80 kV, with samples negatively stained with 2% uranyl acetate prior to imaging. Nanoparticle tracking analysis (NTA, Particle Metrix ZetaView^®^ Evolution, Germany) was used to determine hydrodynamic diameter distribution and particle concentration. Total protein content was measured by Bradford assay (5000201, Bio-Rad, USA), with absorbance at 595 nm recorded on a microplate reader (BioTek Synergy H1, USA).

### 2.4 Triton X-100 treatment of GDNPs

Fresh and dried GDNPs at equal protein concentration were resuspended in 1× PBS and incubated with Triton X-100 (T8200, Solarbio, Beijing, China) at 0.001%, 0.025%, and 0.05% (v/v), or with an equivalent volume of PBS as a negative control, at 37°C for 30 min with gentle shaking. Particle size distribution and concentration were then immediately analyzed by NTA.

### 2.5 Cell culture

Murine macrophage cell line RAW264.7 (CL-0190), human normal colonic epithelial cell line NCM460 (CL-0123), and human colorectal adenocarcinoma cell line Caco-2 (CL-0050) were obtained from Procell Life Technology Co., Ltd (Wuhan, China). Culture media—DMEM (11965092), RPMI-1640 (11875093), MEM (11095080)—together with fetal bovine serum (FBS, 10099141) and penicillin–streptomycin (15140122), were purchased from Gibco (Thermo Fisher Scientific, USA). Cells were maintained at 37°C in a humidified 5% CO2 atmosphere, with medium changes every 48 h.

### 2.6 CCK-8 assay

NCM460 and RAW264.7 cells were seeded in 96-well plates (5 × 10³ cells/well) and treated with GDNPs (0–200 µg/mL) for 24 h to determine the optimal working concentration. Subsequently, cells were treated with GDNPs at the selected concentration for 4, 8, 16, and 24 h, and cell viability was evaluated. The concentrations of GDNPs were normalized based on the total GDNP-associated protein content determined by the Bradford assay. CCK-8 solution (10 µL/well; C0042, Beyotime Biotechnology, Shanghai, China) was added, and the plates were incubated for 30 min in the dark. Optical density was measured at 450 nm using a microplate reader (BioTek Synergy H1, USA).

### 2.7 Western blot

RAW264.7 and NCM460 cells were seeded in 6-well plates (1×10^6^ cells/well). RAW264.7 cells were stimulated with 1 µg/mL LPS (L2630, Sigma-Aldrich, USA) for 6 h, and NCM460 cells with 10 µg/mL LPS plus 40 ng/mL IFN-γ (HY-P7068, MCE, USA) for 24 h, followed by treatment with GDNPs (1, 5, or 10 µg/mL) for 24 h. Cells were lysed in RIPA buffer (P0013B, Beyotime, China) containing protease inhibitors, and protein concentration was determined by Bradford assay. Proteins (30 µg/lane) were separated by 10% SDS-PAGE, transferred to PVDF membranes (IPVH00010, Millipore, USA), and blocked with 5% BSA (A8020, Solarbio, China). Membranes were incubated overnight at 4°C with primary antibodies against TLR4 (AF7017, Affinity, 1:500), Occludin (29275, SAB, 1:1000), Claudin-1 (29226, SAB, 1:1000), p-STAT3 (Tyr705, AF7963, Affinity, 1:500), STAT3 (11045, SAB, 1:1000), and GAPDH (ET1601-4, HUABIO, 1:5000), followed by HRP-conjugated secondary antibody (L3012, SAB, 1:5000) for 1 h at room temperature. Signals were visualized with ECL reagent (S6009M, UElandy) on a ChemiDoc XRS+ imaging system (Bio-Rad).

### 2.8 Trans-epithelial electrical resistance assay

Caco-2 cells (1×10^6^ cells/well) were cultured on 12-well Transwell^®^ inserts (1.2 cm², Corning, USA) with medium renewal every 48 h. TEER was monitored using a Millicell^®^ ERS-2 volt-ohm meter and calculated as TEER (Ω·cm²) = measured resistance (Ω) × membrane area (1.2 cm²). Once TEER exceeded 500 Ω·cm², cells were stimulated with IFN-γ (40 ng/mL) and LPS (10 µg/mL), with or without GDNPs (10 µg/mL), for 24 h. For permeability assessment, FITC-dextran (4 kDa, 60842-46-8, Sigma-Aldrich, USA; 100 µg/mL, 100 µL) was added to the apical chamber for 2 h in the dark, and fluorescence in the basolateral chamber was measured (excitation 485 nm, emission 528 nm). The FITC-dextran extravasation ratio (%) was calculated as: lower-chamber fluorescence/(upper-chamber fluorescence + lower-chamber fluorescence) × 100%.

### 2.9 Dual luciferase reporter assay

NCM460 cells were seeded in 24-well plates (1×10^5^ cells/well) and, at 70–80% confluence, co-transfected using Lipofectamine 3000 (L3000015, Invitrogen, USA) with 200 ng of firefly luciferase reporter plasmid (wild-type or mutant TLR4 3′UTR), 20 ng of *Renilla* luciferase control plasmid (pRL-TK), and 50 nM of miR-29a-5p mimic or negative-control mimic (GenePharma, Shanghai, China). At 30 h post-transfection, cells were lysed in 1× passive lysis buffer, and firefly and *Renilla* luciferase activities were measured sequentially using the Dual-Luciferase Reporter Assay System (E1910, Promega, USA) on a BioTek Synergy H1 microplate reader. Relative luciferase activity was calculated as the firefly/*Renilla* luminescence ratio.

### 2.10 Cell transfection

miR-29a-5p mimics, inhibitors, and corresponding negative controls were obtained from Shanghai GenePharma Biotechnology Co., Ltd. NCM460 and Caco2 cells at 70–80% confluence were transfected with mimics or inhibitors (50 nM) using Hieff Trans^®^ *in vitro* siRNA/miRNA Transfection Reagent (40806ES03, Yeasen, China). After 30 h, GDNPs (final concentration 10 µg/mL) were added for an additional 24 h.

### 2.11 Cell co-culture experiment

To examine macrophage-mediated effects on epithelial cells, RAW264.7 cells were stimulated with LPS, with or without GDNPs, for 24 h, then cultured in serum-free medium for a further 24 h to generate conditioned medium (CM). CM was mixed 1:1 with DMEM and applied to NCM460 cells for 24 h.

### 2.12 Animal experiment

#### 2.12.1 Animal experiment 1: treatment of IBD mice by fresh and dried liquid extract from ginseng

C57BL/6 mice (n=24, 18–20g, 6–8weeks, male) were obtained from Hunan SJA Laboratory Animal Co., Ltd (SCXK 2023-0004). All procedures complied with the Institutional Animal Care and Use Committee guidelines of the Hunan Academy of Chinese Medicine (Ethics Approval No. SY2024-0010).

Mice were randomly divided into 4 groups (n = 6/group): Control (PBS); Model (3% dextran sulfate sodium, DSS, MW 36,000–50,000, Yeasen, Shanghai, China); fresh extract (20 g/kg/d); dried extract (20 g/kg/d). Ginseng was administered by oral gavage from day −7 to day 0, followed by co-administration with 3% DSS drinking water from day 0 to day 7. At the end of the experiment, mice were anesthetized with 3% isoflurane inhalation and euthanized by cervical dislocation. Colon tissues were rapidly collected, flushed with cold PBS, and fixed for histological analysis or stored for molecular assays. The mice were monitored daily for general condition, including activity, food intake, body weight and fecal characteristics.

#### 2.12.2 Animal experiment 2: preventive administration of fresh GDNPs in DSS-induced colitis model

C57BL/6 mice (n=36, 18–20g, 6–8weeks, male)were randomly divided into six groups (n = 6/group): Control (PBS); GDNPs (4×10^11^ particles/mouse, no DSS); Model (3% dextran sulfate sodium, DSS, MW 36,000–50,000, Yeasen, Shanghai, China); SASP (3% DSS + sulfasalazine 0.13 g/kg, MCE, USA); low-concentration GDNPs (LC; 3% DSS + 2×10^11^ particles GDNPs/mouse); and high-concentration GDNPs (HC; 3% DSS + 4×10^11^ particles GDNPs/mouse). GDNPs or SASP were administered by oral gavage from day −7 to day 0, followed by co-administration with 3% DSS drinking water from day 0 to day 7. At the end of the experiment, mice were anesthetized with 3% isoflurane inhalation and euthanized by cervical dislocation. Colon tissues were rapidly collected, flushed with cold PBS, and fixed for histological analysis or stored for molecular assays.

### 2.13 Hematoxylin-eosin staining

Colon tissues were fixed in fresh 4% paraformaldehyde for 24 h at 4°C, dehydrated, and embedded in paraffin. Serial sections (5 µm) were cut, deparaffinized in xylene, rehydrated through a descending alcohol gradient, and rinsed in distilled water before staining with Mayer’s hematoxylin and 0.5% eosin Y according to standard protocols.

### 2.14 Immunohistochemistry

Paraffin-embedded colon sections were subjected to antigen retrieval, endogenous peroxidase blocking, and protein blocking, then incubated with primary antibodies against Occludin (1:500) and Claudin-1 (1:200; AiFang Biological, Changsha, China) and corresponding secondary antibodies. Staining solution was applied and sections were counterstained prior to microscopic observation and imaging.

### 2.15 Immunofluorescence

Colon tissues were fixed in 4% paraformaldehyde, embedded in paraffin and sliced. Section was incubated with primary antibodies (F4/80, CD206, CD86, AiFang biological, Changsha, China) and corresponding secondary antibodies, followed by staining with fluorescent dyes (TYR-570, TYR-520, TYR-650) and DAPI for nuclear counterstaining. Fluorescence images were captured under a fluorescence microscope. Positive areas and positive cells were quantified using ImageJ software (National Institutes of Health, USA).

### 2.16 RT-qPCR

Total RNA was extracted using TRIzol™ Reagent (15596026, Thermo Fisher Scientific, USA). For miRNA analysis, cDNA was synthesized using the miRNA First Strand cDNA Synthesis Kit (MR101-01, Vazyme, China); for mRNA, HiScript^®^ III RT SuperMix (R323-01, Vazyme) was used. qPCR was performed on a LightCycler^®^ 480 II system (Roche, Switzerland) with ChamQ Universal SYBR qPCR Master Mix (Q711-02, Vazyme), in triplicate, under the following conditions: 95°C for 30 s, then 40 cycles of 95°C for 10 s and 60°C for 30 s. Relative expression was normalized to GAPDH (mRNA) or U6 (miRNA) using the 2^-^ΔΔCt method. Primer sequences are provided in **Supplementary Tables S1, S2**.

### 2.17 Statistical analysis

Data are expressed as mean ± SD from at least three independent experiments. Statistical significance was assessed by one-way ANOVA or Student’s *t*-test using GraphPad Prism 5.0 (GraphPad Software, San Diego, CA, USA). *P* < 0.05 was considered statistically significant. Body weight and DAI were monitored daily, while statistical comparisons among groups were performed only at the predefined experimental endpoint using one-way ANOVA followed by Tukey’s multiple-comparison test.

## 3 Results

### 3.1 Comparison of Fresh and dried ginseng extract in IBD model

Previous studies have demonstrated that fresh *Platycodon grandiflorum* confers superior therapeutic efficacy compared with its dried counterpart in LPS-induced acute lung injury (12). Consistently, our results indicated that both fresh and dried ginseng ameliorated DSS-induced body weight loss, decreased DAI scores (**Figures 1A–C**), and inhibited the expression of inflammatory cytokines (**Figures 1F–I**). Notably, fresh ginseng extract showed a greater capacity to improve pathological scores than dried ginseng extract. The dried ginseng group showed more severe inflammation in colonic tissues than the fresh ginseng group, along with a more disorganized glandular arrangement (**Figures 1D, E**).

**Figure 1:**
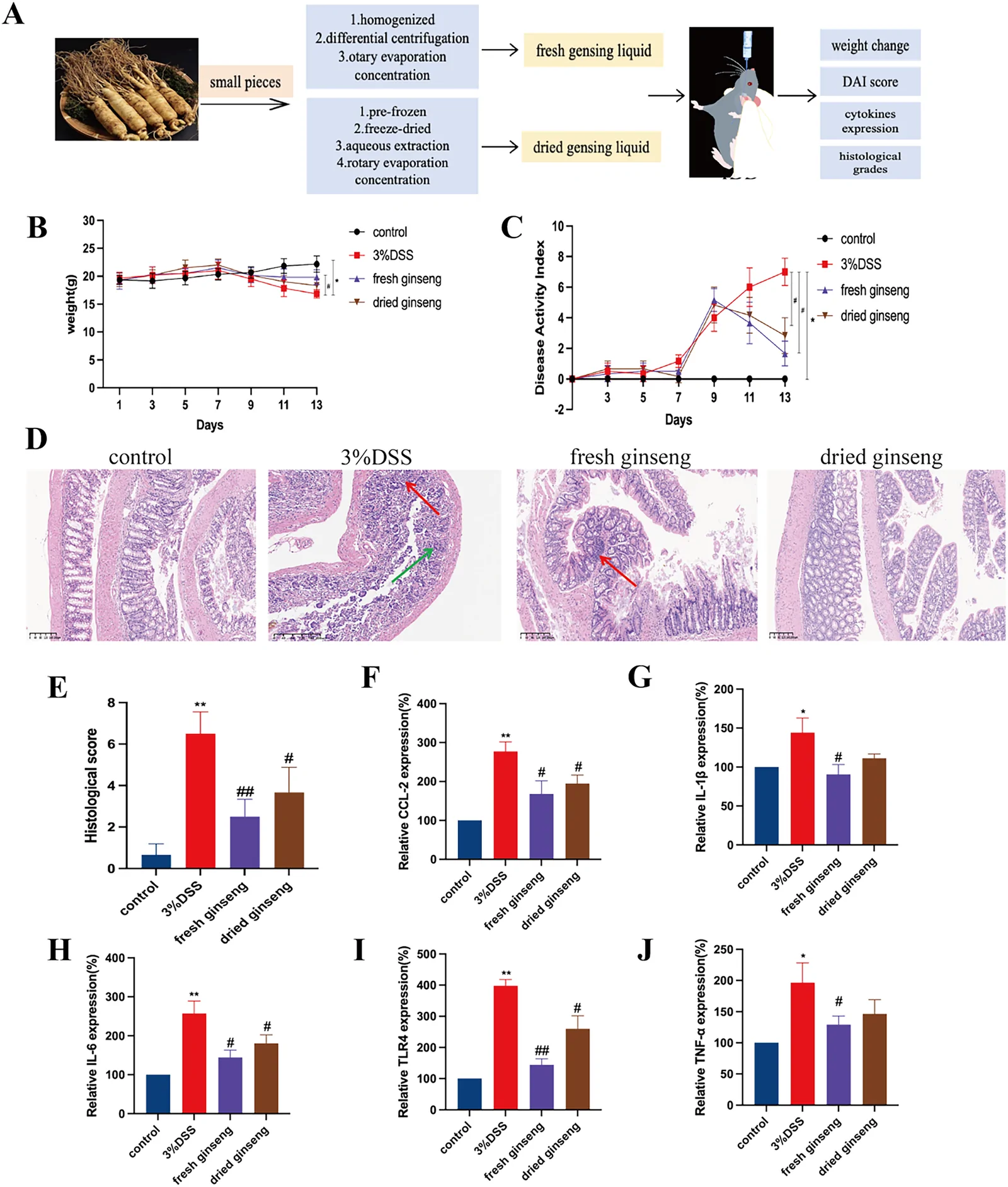
Comparison of fresh and dried ginseng in IBD models. **(A)** Workflow of animal experiments; **(B, C)** Body weight and DAI score; Compared with the control, ^*^indicates *P* < 0.05; Compared with 3% DSS, ^#^indicates *P* < 0.05. **(D, E)** Histopathological score, scale bar: 200µm, the red arrows indicate areas of inflammatory infiltration, and the green arrows indicate crypt damage; **(F–J)**. mRNA expression in colon tissues. Compared with the control, ^*^indicates *P* < 0.05, ^**^indicates *P* < 0.01; Compared with 3% DSS, ^#^indicates *P* < 0.05, ^##^indicates *P* < 0.01.

### 3.2 Isolation and characterization of GDNPs from fresh and dried ginseng

GDNP-enriched vesicle-like nanoparticles were isolated from fresh and dried ginseng root juice by differential centrifugation followed by sucrose density-gradient ultracentrifugation and characterized using multiple complementary approaches, including transmission electron microscopy (TEM) for morphology and nanoparticle tracking analysis (NTA) for particle concentration and size distribution (**Figures 2A–C**). To determine the optimal fraction for fresh GDNPs enrichment, different fractions collected from the sucrose density gradient were analyzed by NTA and TEM. Among the examined fractions, the 30–45% sucrose interface exhibited a higher abundance of nanosized particles with characteristic vesicle-like morphology compared with other fractions, supporting its selection as the fresh GDNP-enriched fraction for subsequent experiments (**Supplementary Figure 1**).

**Figure 2:**
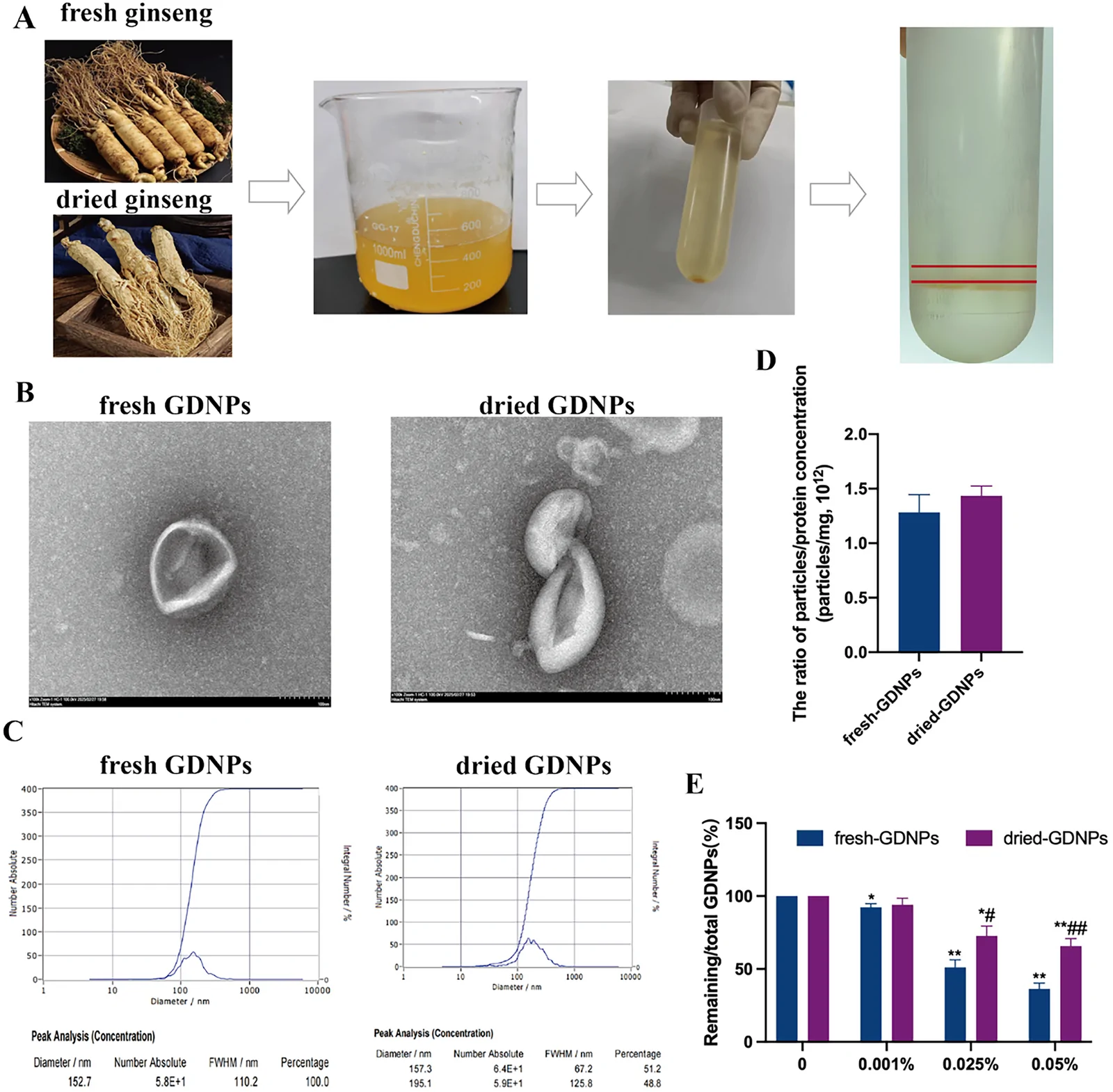
Isolation and characterization of GDNPs from fresh and dried ginseng. **(A)** Isolation and purification of fresh and dried GDNPs; **(B, C)** Morphology and particle size distribution of fresh and dried GDNPs; **(D)** The ratio of particles/protein concentration of fresh and dried GDNPs; **(E)** Remaining/total GDNPs of fresh and dried GDNPs after treatment with Triton X-100. Compared with Triton X-100 0%, ^*^indicates *P* < 0.05, ^**^indicates *P* < 0.01; Compared with the corresponding fresh GDNPs, ^#^indicates *P* < 0.05, ^##^indicates *P* < 0.01.

Both fresh and dried GDNPs displayed a spherical morphology with a bilayer membrane structure and comparable size distributions. To compare relative purity, we used the particle-to-protein concentration ratio together with a Triton X-100 permeabilization assay. No significant difference in particle-to-protein ratio was observed between fresh and dried GDNPs; however, fresh GDNPs underwent Triton X-100-induced lysis to a greater extent, in a concentration-dependent manner, than dried GDNPs (**Figures 2D, E**), suggesting differences in membrane integrity between fresh- and dried-derived GDNP preparations.

### 3.3 Fresh and dried-derived GDNPs show differential epithelial protective activity *in vitro*

To compare the bioactivity of fresh and dried GDNPs on intestinal epithelial cells, NCM460 cells were treated with a range of concentrations of each preparation. Fresh GDNPs promoted cell viability in a concentration- and time-dependent manner, with the maximal effect observed at 10 µg/mL after 24 h (**Figures 3A, B**); in contrast, dried GDNPs at the same concentration had no significant effect on cell viability (**Figures 3C, D**), indicating that fresh GDNPs are more potent promoters of epithelial cell growth. In a Caco-2 model of inflammation-induced barrier dysfunction (LPS + IFN-γ), both fresh and dried GDNPs improved TEER and reduced FITC-dextran permeability relative to the model group, but the protective effect of fresh GDNPs was significantly greater than that of dried GDNPs (**Figures 3E, F**). Both fresh and dried GDNPs could be detected inside NCM460 cells; however, fresh GDNPs exhibited a higher proportion of cellular internalization compared with dried GDNPs (**Figure 3G**), indicating enhanced cellular internalization of fresh GDNPs. These findings suggest that differences in cellular uptake may contribute, at least partially, to the differential epithelial protective effects observed between fresh and dried GDNPs. Based on these results, fresh GDNPs were selected for the subsequent mechanistic studies of intestinal barrier protection and anti-inflammatory activity in IBD.

**Figure 3:**
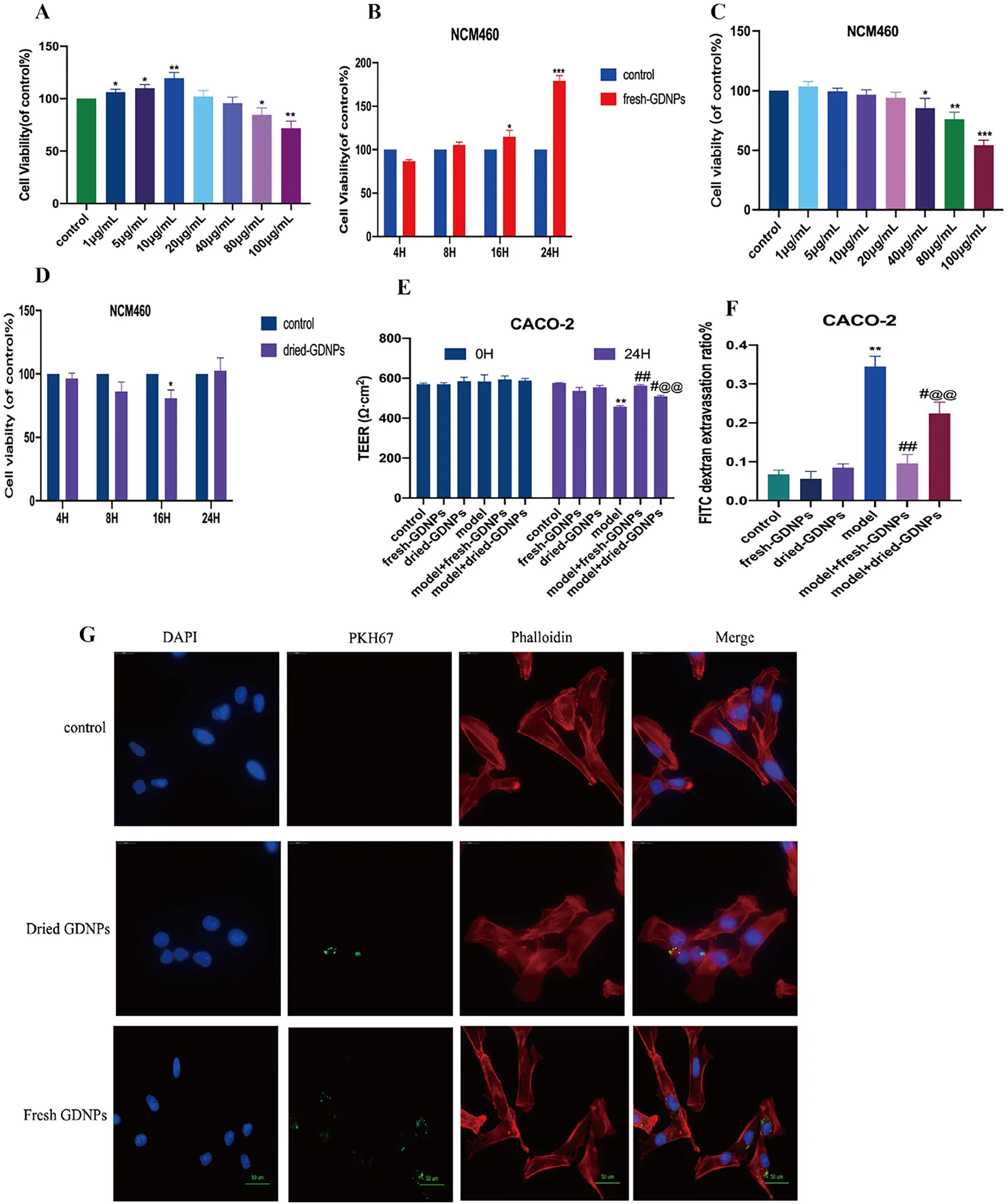
Comparison of fresh and dried GDNPs in cell viability and cellular permeability. **(A)** NCM460 cells were treated with different concentrations of fresh GDNPs for 24 h, was used to assess cell viability; Compared with control, ^*^indicates *P* < 0.05, ^**^indicates *P* < 0.01. **(B)** NCM460 cells were treated with 10 μg/mL of fresh GDNPs for 4–24 h, CCK8 was applied to detect the cell viability; Compared with the corresponding control at each time point, ^*^indicates *P* < 0.05, ^***^indicates *P* < 0.001. **(C)** NCM460 were treated with different concentrations of dried GDNPs for 24 h, CCK8 was applied to detect the cell viability; Compared with control, ^*^indicates *P* < 0.05, ^**^indicates *P* < 0.01, ^***^indicates *P* < 0.001. **(D)** NCM460 were treated with 10 μg/mL of dried GDNPs for 4–24 h, CCK8 was applied to detect the cell viability; Compared with the corresponding control at each time point, ^*^indicates *P* < 0.05. **(E, F)**. CACO-2 cells were stimulated with IFN-γ (40 ng/mL) and LPS (10 μg/mL), and then treatment with either fresh or dried GDNPs (10 μg/mL) for 24 h; the transepithelial electrical resistance (TEER) and FITC-dextran permeability were measured. Compared with the control, ^*^indicates *P* < 0.05; Compared with the model, ^#^indicates *P* < 0.05, ^##^indicates *P* < 0.01; Compared with the model+fresh-GDNPs, ^@@^indicates *P* < 0.01; **(G)** Cellular uptake of dried and fresh ginseng in NCM460.

### 3.4 Fresh GDNPs preserve the epithelial barrier by restoring tight junction proteins and miR-29a-5p while suppressing inflammatory mediators

In LPS/IFN-γ-stimulated NCM460 cells, western blotting showed that fresh GDNPs suppressed TLR4/STAT3 activation in a dose-dependent manner across low (1 µg/mL), middle (5 µg/mL), and high (10 µg/mL) concentrations, and reversed the inflammation-induced downregulation of Occludin and Claudin-1 (**Figures 4A–E**). RT-qPCR showed that high-concentration GDNPs effectively suppressed pro-inflammatory cytokine mRNA expression (IL-6, IL-1β, TNF-α) (**Figure 4F**). Based on a literature survey, we selected three miRNAs implicated in epithelial barrier homeostasis (miR-29a-5p, miR-125b-5p, and miR-21-5p) for further analysis. All three were downregulated by inflammatory stimulation; high-concentration GDNPs robustly restored miR-29a-5p and miR-21-5p expression, whereas miR-125b-5p remained unaffected (**Figure 4G**). Given its consistent responsiveness and established link to TLR4 signaling, miR-29a-5p was selected for further mechanistic investigation.

**Figure 4:**
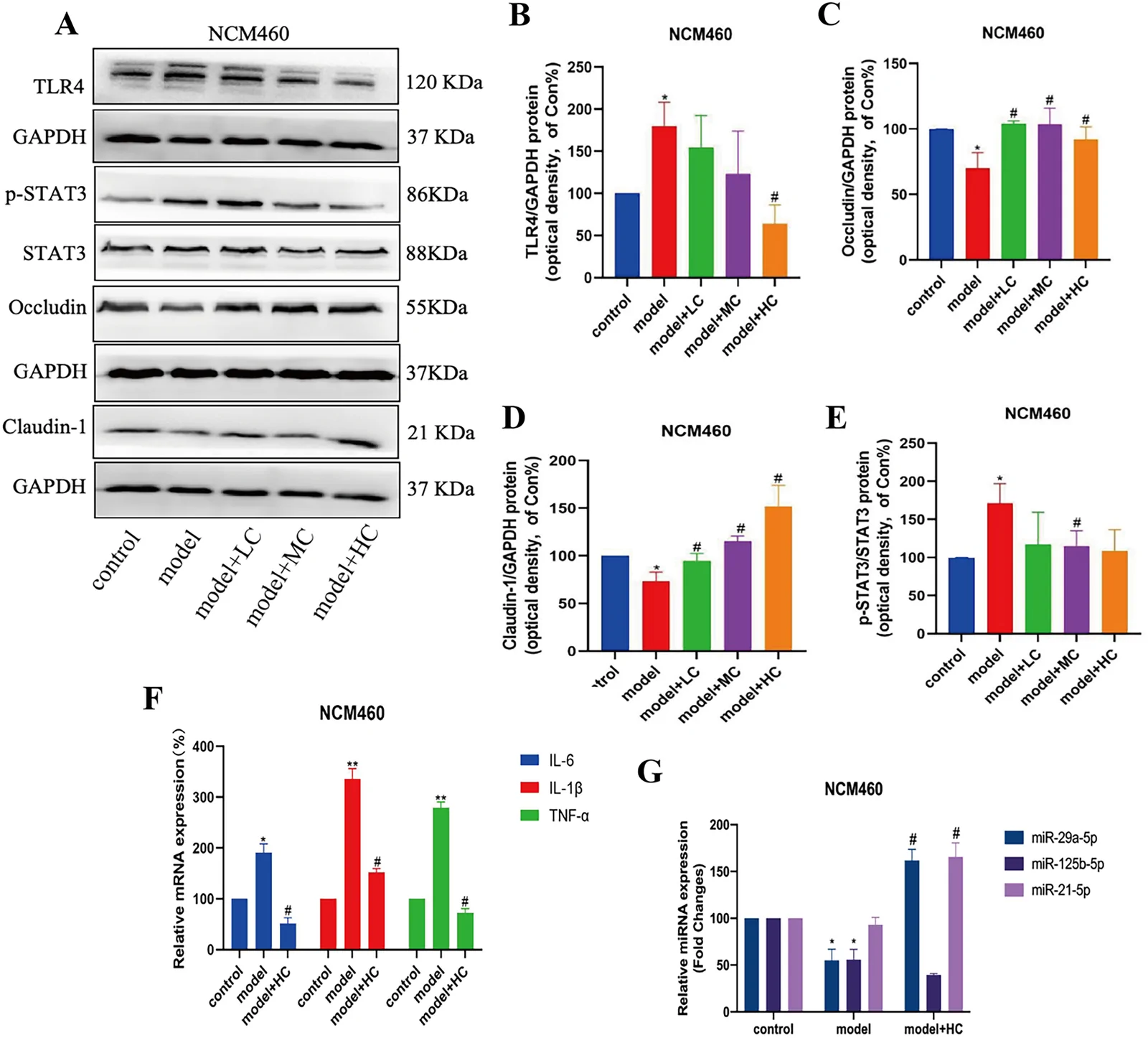
GDNPs preserve the epithelial barrier by modulating the expression tight junction proteins, inflammation factor and miR-29a-5p. **(A–E)**. NCM460 cells were stimulated with IFN-γ (40 ng/mL) and LPS (10 μg/mL), and then treatment with low, middle and high dose of fresh GDNPs (1, 5 and 10 μg/mL) for 24 h, western blot was used for detecting proteins expression; Compared with the control, ^*^indicates *P* < 0.05; Compared with the model, ^#^indicates *P* < 0.05. **(F, G)** NCM460 cells were stimulated with IFN-γ (40 ng/mL) and LPS (10 μg/mL), and then treated with fresh GDNPs (10 μg/mL) for 24 h, RT-qPCR was applied for detecting cytokines and miRNAs expression. Compared with the control, ^*^indicates *P* < 0.05, ^**^indicates *P* < 0.01; Compared with the model, ^#^indicates *P* < 0.05.

### 3.5 Fresh GDNPs protect the epithelial barrier through miR-29a-5p-mediated TLR4 suppression

Given previous evidence that miR-29a overexpression attenuates cholestatic liver fibrosis through TLR4 suppression (13), we examined this regulatory axis in the intestinal epithelium. In TEER assays, miR-29a-5p mimic transfection partially restored LPS/IFN-γ-induced barrier dysfunction and acted synergistically with GDNPs to further improve barrier function (**Figures 5A, B**). Conversely, miR-29a-5p knockdown in NCM460 cells increased TLR4 expression and decreased Occludin and Claudin-1 expression, effects that were significantly reversed by GDNP treatment (**Figures 5C–F**). These findings suggest that miR-29a-5p contributes to GDNP-mediated regulation of epithelial barrier integrity. Bioinformatic prediction identified conserved miR-29a-5p binding sites within the TLR4 3′UTR, and dual-luciferase reporter assays confirmed direct targeting of TLR4 by miR-29a-5p (**Figure 5G**). Collectively, these data indicate that the miR-29a-5p/TLR4 signaling pathway is involved in the protective effects of GDNPs on epithelial barrier function.

**Figure 5:**
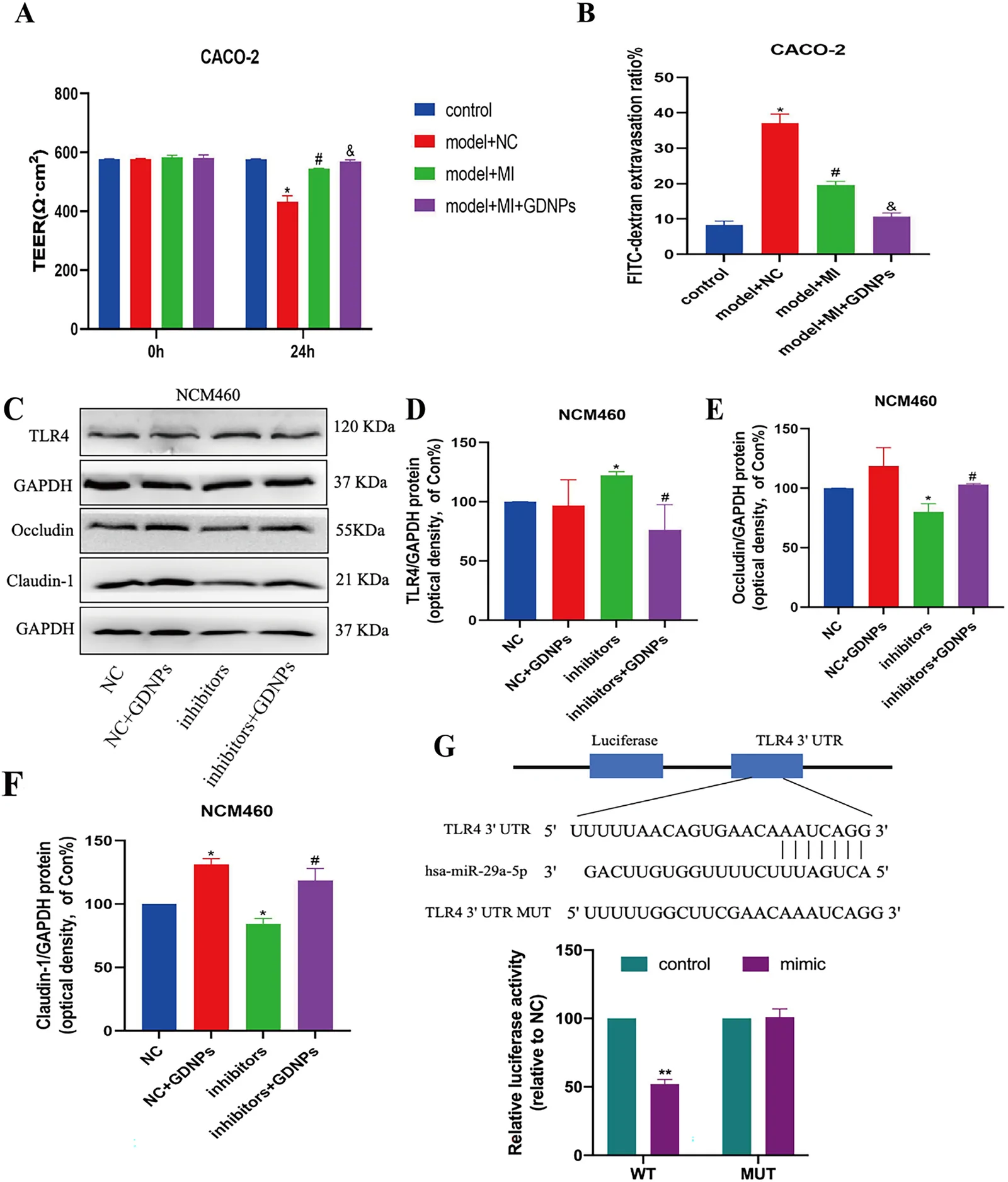
GDNPs protect the epithelial barrier by regulating miR-29a-5p-mediated TLR4 downregulation. **(A, B)** CACO-2 cells were stimulated with IFN-γ (40 ng/mL) and LPS (10 μg/mL), then transfected with miR-29a-5p mimics (50 nM) for 30 h, fresh GDNPs (10 μg/mL) was added to the culture system for an additional 24 h, transepithelial electrical resistance (TEER) and FITC-dextran permeability were measured; Compared with the control, ^*^indicates *P* < 0.05; Compared with the model, ^#^indicates *P* < 0.05; Compared with the model+MI, ^&^indicates *P* < 0.05. **(C–F)** NCM460 cells were transfected with miR-29a-5p inhibitors (50 nM) for 30 h, GDNPs (10 μg/mL) was added to the culture system for an additional 24 h, western blot was applied for detecting proteins expression; Compared with the NC, ^*^indicates *P* < 0.05; Compared with the inhibitors, ^#^indicates *P* < 0.05. **(G)** NCM460 cells were transfected with TLR4 3’UTR and miR-29a-5p mimics (50 nM) for 30 h, dual luciferase assay was applied for detecting fluorescence value; Compared with the NC, ^**^indicates *P* < 0.01.

### 3.6 Fresh GDNPs protect the epithelial barrier by modulating macrophage–epithelial crosstalk

We next examined the effects of GDNPs on RAW264.7 macrophages. Treatment with 1–40 µg/mL GDNPs promoted macrophage viability, with 1 µg/mL eliciting the most pronounced effect after 24 h (**Figures 6A, B**). In LPS-stimulated macrophages, GDNPs dose-dependently attenuated the LPS-induced upregulation of TLR4 protein (**Figures 6C, D**) and suppressed the mRNA expression of IL-6, CCL-2, and TNF-α while restoring miR-29a-5p expression, with the most pronounced effects observed at the high concentration (**Figures 6E, F**).

**Figure 6:**
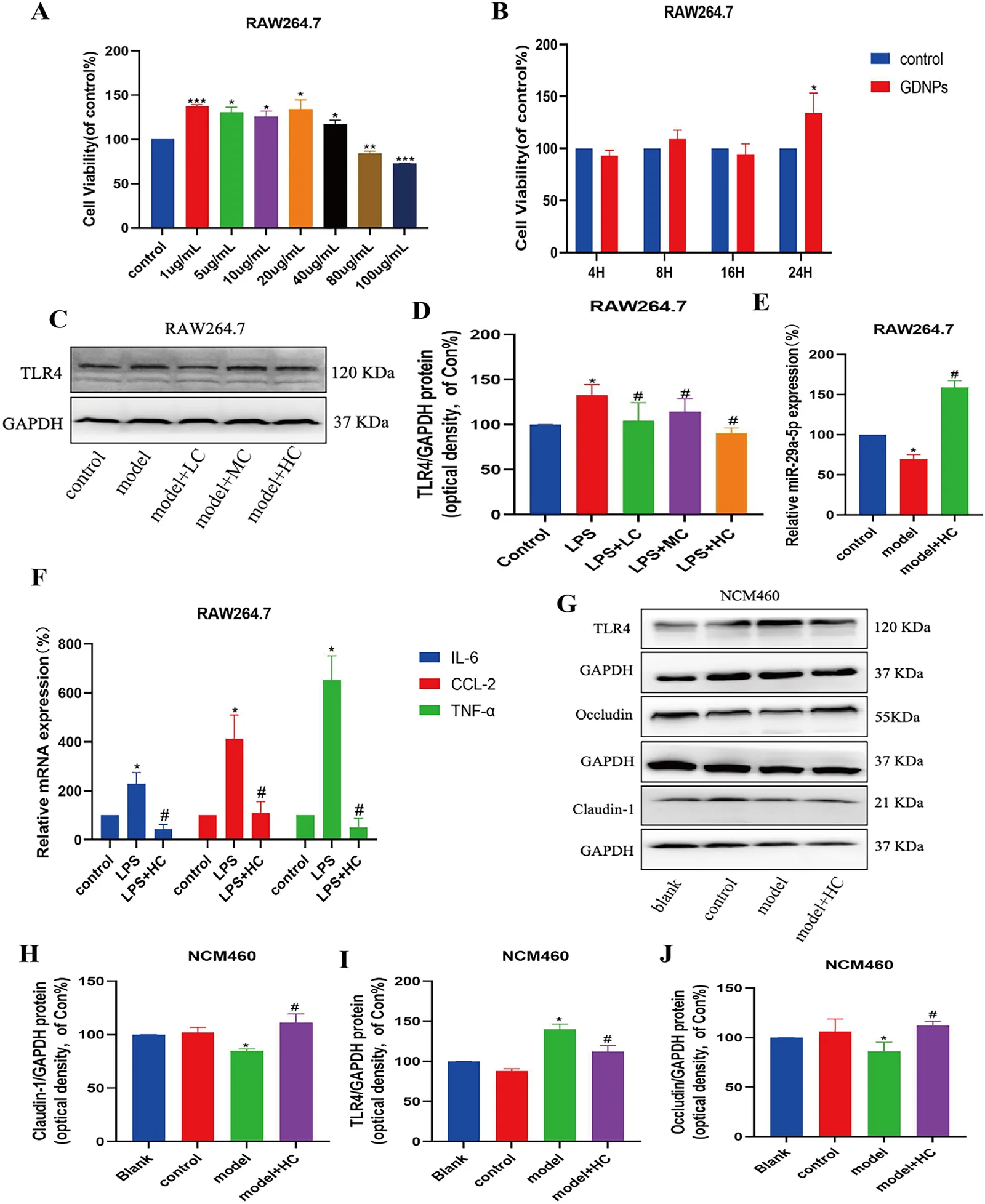
GDNPs protect the epithelial barrier by regulating crosstalk between macrophage and colonic epithelial cells. **(A, B)** RAW264.7 cells were treated with different concentrations of fresh GDNPs for 24 h, CCK8 was used for measuring the cell viability; **(B)** RAW264.7 cells were treated with 1 μg/mL of fresh GDNPs for 4–24 h, CCK8 was applied to detect the cell viability; Compared with the control, ^*^indicates *P* < 0.05, ^**^indicates *P* < 0.01, ^***^indicates *P* < 0.001; **(C, D)** RAW264.7 cells were stimulated with LPS (1 μg/mL), and then treated with low, middle and high dose of fresh GDNPs (1, 5 and 10 μg/mL) for 24 h, western blot was applied for detecting proteins expression; Compared with the control, ^*^indicates *P* < 0.05; Compared with the model, ^#^indicates *P* < 0.05. **(E, F)** RAW264.7 cells were stimulated with LPS (1 μg/mL), and then treated with fresh GDNPs (10 μg/mL) for 24 h, RT-qPCR was applied for detecting cytokines and miR-29a-5p expression; Compared with the control, ^*^indicates *P* < 0.05; Compared with the model, ^#^indicates *P* < 0.05. **(G–J)** NCM460 cells were cultured with RAW264.7 conditional medium (control, model and model+HC) for 24 h, western blot was applied for detecting proteins expression. Compared with the control, ^*^indicates *P* < 0.05; Compared with the model, ^#^indicates *P* < 0.05.

To assess whether GDNPs-modified macrophage secretory products affect epithelial barrier integrity, NCM460 cells were exposed to conditioned medium (CM) from differentially treated RAW264.7 macrophages. CM from LPS-stimulated macrophages (LPS-CM) increased TLR4 expression and decreased Occludin and Claudin-1 expression in NCM460 cells, whereas CM from GDNPs-co-treated macrophages (GDNPs-CM) suppressed TLR4 and restored tight junction protein expression (**Figures 6G–J**). Consistently, LPS-CM decreased miR-29a-5p expression in NCM460 cells, an effect reversed by GDNPs-CM (**Figure 6K**). These findings suggest that macrophage-associated paracrine communication may contribute to fresh GDNPs-mediated epithelial protection.

### 3.7 Preventive administration of fresh GDNPs ameliorates DSS-induced intestinal inflammation and pathological injury

Fresh GDNPs were subjected to sequential simulated gastric (SGF, pH 2.0) and intestinal (SIF, pH 6.8) digestion to evaluate gastrointestinal stability. TEM analysis demonstrated that GDNPs retained recognizable vesicle-like structures after digestion, although mild morphological changes were observed. NTA analysis showed that SGF treatment reduced detectable particle concentration, whereas subsequent SIF treatment slightly increased particle numbers (**Supplementary Figure 2**), suggesting dynamic structural remodeling during digestion. Importantly, digested GDNPs retained their ability to promote colon epithelial cell viability, suppress inflammatory cytokine production, and preserve tight junction proteins (**Supplementary Figure 3**), indicating functional preservation after simulated gastrointestinal transit.

To define an effective dosing range, five doses of GDNPs (1×10^11^, 2×10^11^, 4×10^11^, 8×10^11^, and 1.6×10^12^ particles/mouse) were administered by oral gavage to healthy mice. The two highest doses (8×10^11^ and 1.6×10^12^ particles/mouse) caused significant weight loss, whereas the intermediate doses (2×10^11^ and 4×10^11^ particles/mouse) promoted weight gain, and the lowest dose (1×10^11^ particles/mouse) had no significant effect on body weight. Based on these findings, 2×10^11^ (low concentration, LC) and 4×10^11^ (high concentration, HC) particles/mouse were selected for subsequent preventive efficacy studies.

Using a 3% DSS-induced colitis model with sulfasalazine (SASP) as a positive control (**Figure 7A**), we found that HC GDNPs and SASP both significantly attenuated DSS-induced body weight loss, preserved colon length, and reduced DAI scores (**Figures 7B–E**). Notably, HC GDNPs alone (without DSS) modestly increased body weight in healthy mice but had no measurable effect on DAI scores or colon morphology, suggesting that the barrier-protective and growth-promoting effects of GDNPs are not associated with overt toxicity. Histologically, DSS induced characteristic colonic damage, including goblet cell depletion, inflammatory cell infiltration between crypts, and disrupted mucosal architecture; both SASP and HC GDNPs treatment restored goblet cell populations, reduced inflammatory infiltration, and improved mucosal integrity (**Figures 7F, G**).

**Figure 7:**
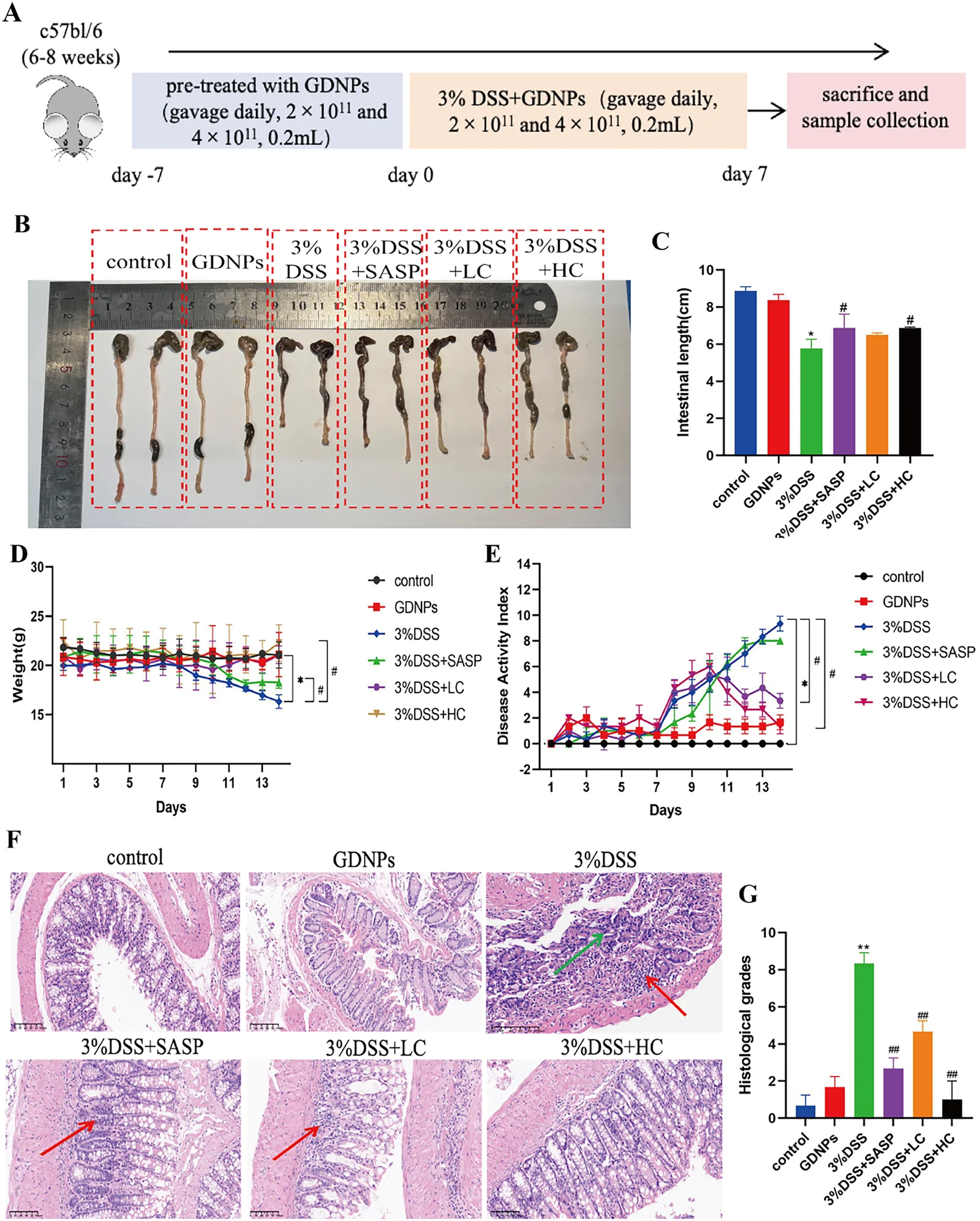
GDNPs ameliorate intestinal inflammation and pathological injury in IBD models. **(A)** Timeline of animal experiments; **(B, C)** Colonic morphology and length; **(D)**. Body weight; **(E)** DAI score; Compared with the control, ^*^indicates *P* < 0.05; Compared with 3% DSS, ^#^indicates *P* < 0.05. **(F, G)**. Histopathological score, scale bar: 100µm, the red arrows indicate areas of inflammatory infiltration, and the green arrows indicate crypt damage.

### 3.8 Fresh GDNPs modulate macrophage inflammatory phenotype and preserve tight junction protein expression

Pro-inflammatory macrophage activation contributes to intestinal inflammation and barrier dysfunction in IBD (14). Immunofluorescence analysis showed that DSS administration increased the proportion of CD86^+^ macrophages in colonic tissues, whereas GDNP treatment reduced CD86 expression and increased CD206 expression compared with the DSS group (**Figures 8A, B**); suggesting that GDNPs modulate macrophage inflammatory phenotypes in the inflamed colon. DSS administration also caused substantial downregulation of Occludin and Claudin-1, which was significantly reversed by SASP or HC GDNPs treatment, with expression levels approaching those of healthy controls (**Figures 8C–F**). These findings indicate that GDNPs alleviate intestinal barrier injury *in vivo* and may contribute to barrier protection through regulation of macrophage inflammatory responses and preservation of epithelial tight junction integrity.

**Figure 8:**
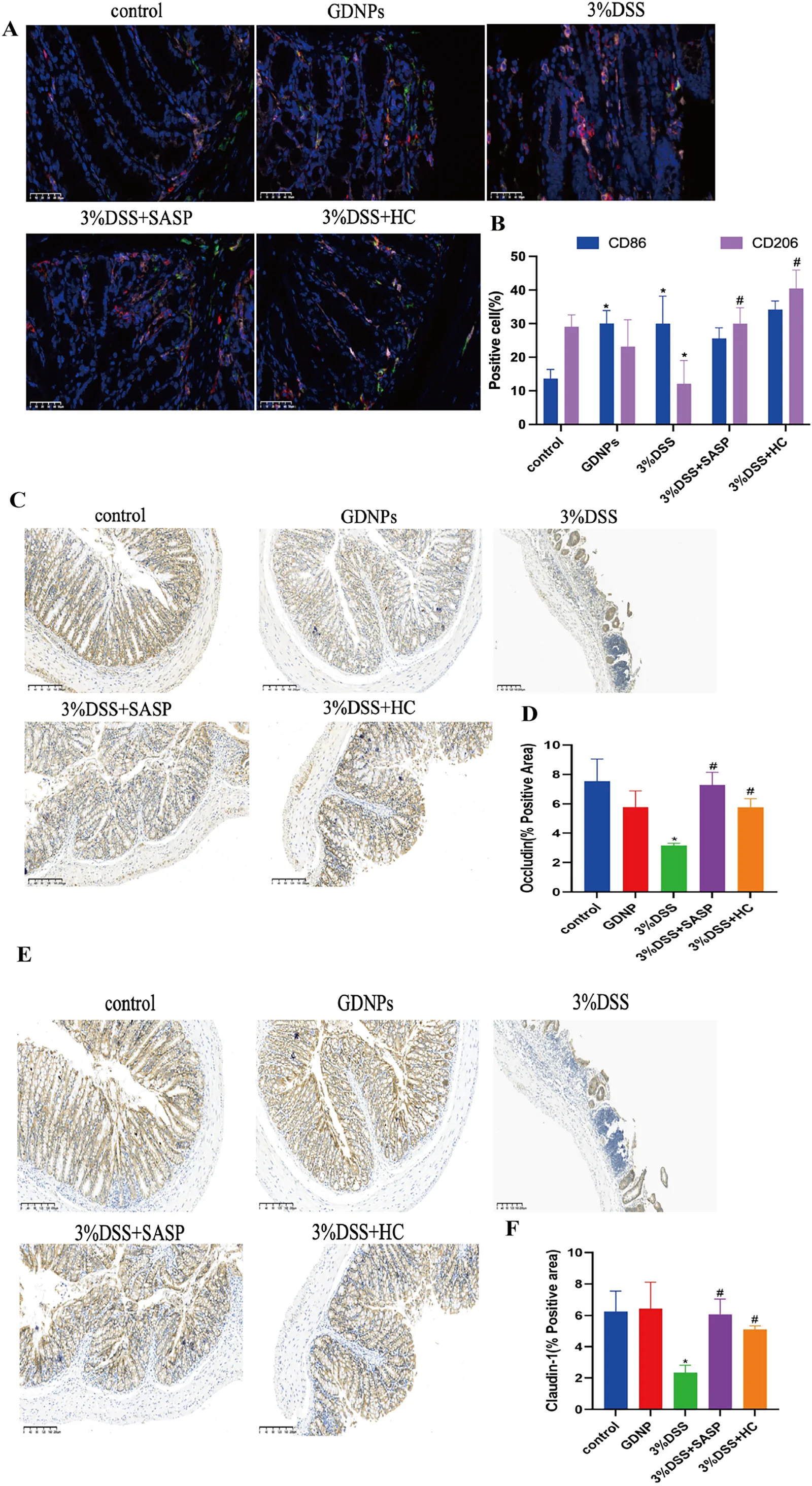
GDNPs decrease M1 macrophage polarization and protect the intestinal epithelial barrier in IBD models. **(A, B)** Immunofluorescence was applied to evaluate macrophage polarization (20×); CD86 (red), CD206 (green), DAPI (blue), F4/80 (pink), scale bar: 50µm. **(C–F)**. Immunohistochemical was applied to test tight junction proteins expression, scale bar: 200µm; Compared with the control, ^*^indicates *P* < 0.05; Compared with 3% DSS, ^#^indicates *P* < 0.05.

### 3.9 Fresh GDNPs suppress inflammatory mRNA expression and restore miR-29a-5p expression *in vivo*

RT-qPCR showed that DSS markedly upregulated colonic mRNA levels of IL-6, IL-1β, TNF-α, CCL-2, and TLR4 (**Figures 9A–E**), consistent with the increased M1 polarization observed above. Both SASP and GDNPs (LC and HC) effectively counteracted these DSS-induced changes. In addition, DSS administration significantly downregulated miR-29a-5p expression in colon tissue, an effect reversed by SASP and HC GDNPs treatment (**Figure 9F**), corroborating the *in vitro* findings that GDNPs act through restoration of miR-29a-5p.

**Figure 9:**
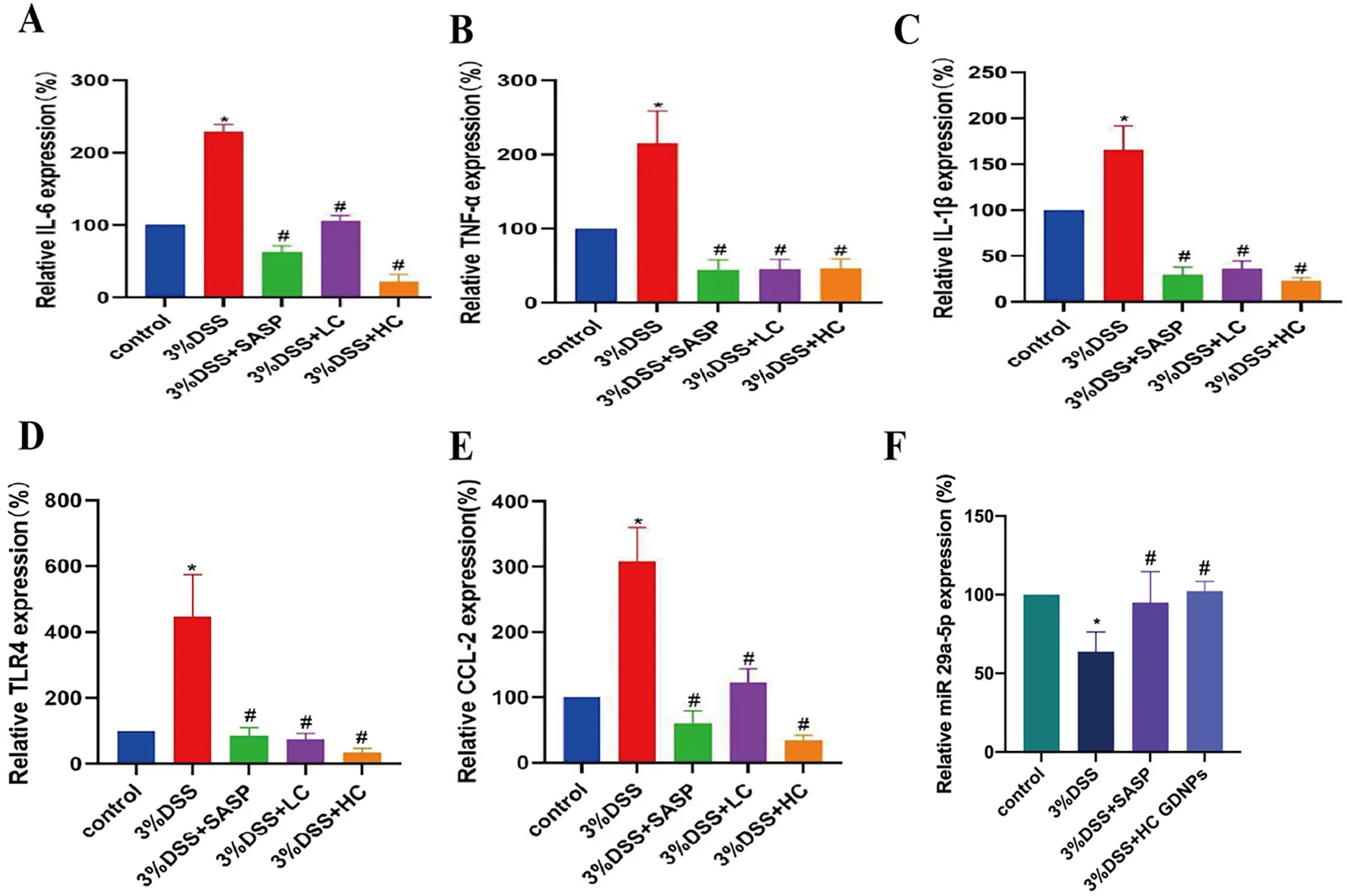
GDNPs suppress inflammation-related mRNA expression and enhance miR-29a-5p expression in IBD models. **(A–E)**. RT-qPCR was applied to detect IL-6, IL-1β, TNF-α, CCL-2 and TLR4 mRNA expression in colon tissue; **(F).** RT-qPCR was applied to detect miR-29a-5p expression in colon tissue; Compared with the control, ^*^indicates *P* < 0.05; Compared with 3% DSS, ^#^indicates *P* < 0.05.

## 4 Discussion

Ginseng (*Panax ginseng* C.A. Meyer) is a traditional Chinese herbal medicine with well-documented benefits for digestive disorders, including IBD (15, 16). Beyond its bioactive small molecules, ginseng has more recently been recognized as a source of GDNPs—naturally derived nanovesicles with therapeutic activity in glioblastoma (17), UV-induced cutaneous photodamage (18), wound healing (19), and ethanol-induced hepatic injury (20). Comprehensive lipidomic and proteomic profiling has identified 23 lipid subclasses (predominantly diacylglycerols and phosphatidic acids) and 202 unique proteins within GDNPs, many of which are functionally linked to MAPK signaling (9), while quantification of ginsenoside Rg3 has demonstrated batch-to-batch consistency, supporting the pharmaceutical standardization potential of GDNPs preparations (21).

A key translational question for any plant-derived nanoparticle therapeutic is whether the choice of raw material influences nanoparticle quality and bioactivity. However, this aspect remains insufficiently explored in GDNP research, as previous IBD studies have primarily focused on GDNPs derived from fresh ginseng (9–11). Here, we show that fresh and dried ginseng yield GDNPs with similar morphology and particle-to-protein ratios, but fresh-derived GDNPs showed different detergent sensitivity and stronger epithelial protective activity *in vitro*, suggesting that processing-related changes may influence nanoparticle integrity and biological activity. Importantly, the *in vivo* comparison between fresh and dried ginseng extracts reflects differences in the overall biological activity of whole ginseng preparations, which may involve multiple bioactive components. The *in vitro* comparison of isolated GDNPs further suggests that differences in GDNPs properties may contribute to, but cannot fully account for, the functional differences observed between fresh and dried preparations. A similar advantage of fresh over dried plant material has been reported for *Platycodon grandiflorum* in acute lung injury (22), where EVs derived from fresh *Platycodon grandiflorum* were shown to contribute to the observed protective effects. This suggests that raw-material-dependent variability may represent a general feature of plant exosome-like nanoparticles and should be carefully considered in future manufacturing and quality-control strategies. In particular, standardized post-harvest processing and compositional fingerprinting may be more appropriate than reliance on morphology- or protein-based characterization alone.

Building on this raw-material comparison, our mechanistic studies clarify how fresh GDNPs protect the intestinal barrier. LPS-induced inflammation is a well-established driver of epithelial barrier injury (23), and tight junction proteins such as occludin and claudin family members govern paracellular permeability between the luminal and serosal compartments (24). We found that fresh GDNPs suppressed LPS-induced macrophage inflammation (lower IL-6, IL-1β, TNF-α, and TLR4) while strengthening the colonic epithelial barrier (higher Occludin and Claudin-1). Notably, conditioned medium from fresh GDNP-treated macrophages was sufficient to protect epithelial permeability, indicating that fresh GDNPs exert their barrier-protective effects not only through direct actions on epithelial cells but also indirectly through modulation of macrophage-mediated paracrine communication. While macrophage–epithelial crosstalk has been primarily studied in inflammatory injury, such as *Helicobacter pylori*-induced disruption of gastric mucosal homeostasis through macrophage-derived CCL3 (25), our findings suggest that this communication axis can also be therapeutically modulated to promote intestinal barrier repair.

Several miRNAs, including miR-29a (26, 27), miR-125b-5p (28), and miR-21-5p (29), have been implicated in intestinal barrier regulation. We found that all three were downregulated by inflammatory stimulation, but only miR-29a-5p and miR-21-5p were consistently restored by fresh GDNPs, and this pattern was reproduced *in vivo* for miR-29a-5p, suggesting that miR-29a-5p may contribute to fresh GDNP-mediated intestinal barrier protection. Mechanistically, miR-29a-5p knockdown activated TLR4 signaling and suppressed Occludin and Claudin-1, consistent with previous work showing that miR-29a attenuates obstructive jaundice-induced fibrosis via TLR2/4 suppression (27). Our dual-luciferase data extend this relationship by directly demonstrating that miR-29a-5p binds the TLR4 3′UTR in intestinal epithelial cells. These findings, together with previous studies showing that TLR4 deficiency preserves tight junction protein expression and intestinal barrier function during LPS-induced sepsis (30), suggest that modulation of the miR-29a-5p/TLR4 signaling pathway may be involved in the barrier-protective effects of fresh GDNPs.

This study has several limitations. First, although fresh and dried GDNPs showed differential epithelial protective effects *in vitro*, their direct comparison *in vivo* was not performed; therefore, whether post-harvest processing alters the *in vivo* activity of GDNPs requires further investigation. Second, our findings suggest that miR-29a-5p/TLR4 signaling may contribute to the protective effects of GDNPs; however, GDNPs contain diverse bioactive cargos, and the relative contribution of miR-29a-5p and other components remains to be clarified. Third, the molecular basis underlying the functional differences between fresh and dried GDNPs, including potential alterations in membrane composition and cargo profiles after processing, requires further investigation. Finally, the DSS model does not fully recapitulate the complexity of human IBD, and additional studies involving biodistribution analysis, long-term safety evaluation, and chronic colitis models are needed to facilitate clinical translation.

## 5 Conclusion

In conclusion, this study demonstrates that post-harvest processing may influence the functional properties of GDNPs, while fresh ginseng-derived GDNPs protect intestinal barrier integrity in DSS-induced colitis through involvement of the miR-29a-5p/TLR4 pathway and macrophage–epithelial communication.

## Data Availability

The raw data supporting the conclusions of this article will be made available by the authors, without undue reservation.
