# Supplementary material for: Fresh ginseng-derived vesicle-like nanoparticles protect against intestinal barrier injury in inflammatory bowel disease via the miR-29a-5p/TLR4 axis
Source: Front Immunol. 2026 Sep 30;17:1929376. doi: 10.3389/fimmu.2026.1929376 (PMC13661198; doi:10.3389/fimmu.2026.1929376)
Supplement: Supplementary file 1 [file Table1.docx]

Table 1 Primers used for the qRT-PCR study (Homo sapiens)

| Gene | Sequence (5'-3') |
| --- | --- |
| GAPDH-F | AATTCCATGGCACCGTCAAG |
| GAPDH-R | AGCATCGCCCCACTTGATTT |
| IL-6-F | TTCGGTCCAGTTGCCTTCTC |
| IL-6-R | CTGAGATGCCGTCGAGGATG |
| IL-1β-F | AAGTACCTGAGCTCGCCAGT |
| IL-1β-R | CTTGCTGTAGTGGTGGTCGG |
| TNF-α-F | CCCAGGGACCTCTCTCTAATC |
| TNF-α-R | ATGGGCTACAGGCTTGTCACT |
| miR-29a-5p-F | AGCACCAUCUGAAAUCGGUUA |
| miR-29a-5p-R | GTGCAGGGTCCGAGGT |
| U6-F | CTCGCTTCGGCAGCACA |
| U6-R | AACGCTTCACGAATTTGCGT |
| miR-29a-5p NC sense | UUCUCCGAACGUGUCACGUTT |
| miR-29a-5p NC antisense | ACGUGACACGUUCGGAGAATT |
| miR-29a-5p inhibitor | CUGAACACCAAAAGAAAUCAGU |
| miR-29a-5p mimics sense | ACUGAUUUCUUUUGGUGUUCAG |
| miR-29a-5p mimics antisense | GAACACCAAAAGAAAUCAGUUU |

F: forward; R: reverse

Table 2 Primers used for the qRT-PCR study (Mus musculus)

| Gene | Sequence (5'-3') |
| --- | --- |
| GAPDH-F | AAGGTCATCCCAGAGCTGAA |
| GAPDH-R | CTGCTTCACCACCTCTTGA |
| IL-6-F | CGGAGAGGAGACTTCACAGAG |
| IL-6-R | CATTTCCACGATTTCCCAGA |
| CCL-2-F | CACTCACCTGCTGCTACTCATTC |
| CCL-2-R | GGTGCTGAAGACCTTAGGGC |
| TNF-α-F | TTGTCTACTCCCAGGTTCTCT |
| TNF-α-R | GAGGTTGACTTTCTCCTGGTATG |
| miR-29a-5p-F | GCGCGACTGATTTCTTTTGGT |
| miR-29a-5p-R | AGTGCAGGGTCCGAGGTATT |
| miR-125b-5p-F | ACTGATAAATCCCTGAGACCCTAAC |
| miR-125b-5p-R | TATGGTTTTGACGACTGTGTGAT |
| miR-21-5p-F | ACACTCCAGCTGGGTAGCTTATCAGACTGA |
| miR-21-5p-R | GTGTCGTGGAGTCGGCAATTC |
| U6-F | AGAGAAGATTAGCATGGCCCCTG |
| U6-R | AGTGCAGGGTCCGAGGTATT |

F: forward; R: reverse
